# Do high house prices promote the development of China’s real economy? Empirical evidence based on the decomposition of real estate price

**DOI:** 10.1371/journal.pone.0295311

**Published:** 2024-01-11

**Authors:** Wei Fan, Yun He, Liang Hao, Fan Wu

**Affiliations:** 1 School of Politics and Public Administration, Zhengzhou University, Zhengzhou, China; 2 Finance School, Hubei University of Economics, Wuhan, China; 3 School of Economics and Management, TianShi College, Tianjin, China; 4 The Postdoctoral Workstation of Shenzhen Capital Group, Shenzhen Capital Group, Shenzhen, China; Guangdong University of Foreign Studies, CHINA

## Abstract

Moderate rising of house prices are beneficial to the economic development. However, over high house prices worsen the economic distortions and thus hinder the development of the real economy. We use the stochastic frontier models to calculate the fundamental value in the housing in Chinese large and medium cities, and then obtain indexes which could measure the house prices’ deviations from the fundamental value. With the macroeconomic data in the city-level, this paper empirically investigates the effects of the house prices’ deviations on macro-economic variables like consumption, investment and output. The study reveals that the housing bubble exists in most Chinese cities, and first-tier cities fare the worst. House prices over the fundamental value, which could increase the scale of real estate investment, bring adverse impacts on GDP, as it causes declining civilian consumption and discourages real economy’s investment and production. The encouragement and the discouragement on macroeconomy caused by house prices’ deviation from its basic value take turns to play a key role in the process of China’ eco-nomic growth. In the early stage of China’s economic growth, the encouragement effect predominates. As urbanization and industrialization gradually upgrade to a higher level, the discouragement effect takes charge.

## 1. Introduction

Does the real estate bubble promote or inhibit the growth of the macroeconomy? To answer this question, this paper employs cutting-edge econometric methods to quantify China’s real estate bubble and uses empirical research to investigate its specific impacts on macroeconomic growth. The real estate market plays a pivotal role in macroeconomics, influencing national and regional economic growth and stability. It’s also crucial for the residential well-being and wealth accumulation of millions of households. However, with urbanization and economic development, the real estate market faces numerous challenges. Especially in some of China’s major cities and economically developed areas, an excessively high real estate bubble has resulted in residents bearing heavy housing costs, declining consumption levels, and imbalances in market resource allocations. This has drawn widespread attention from both academia and society.

**Fig 1:**
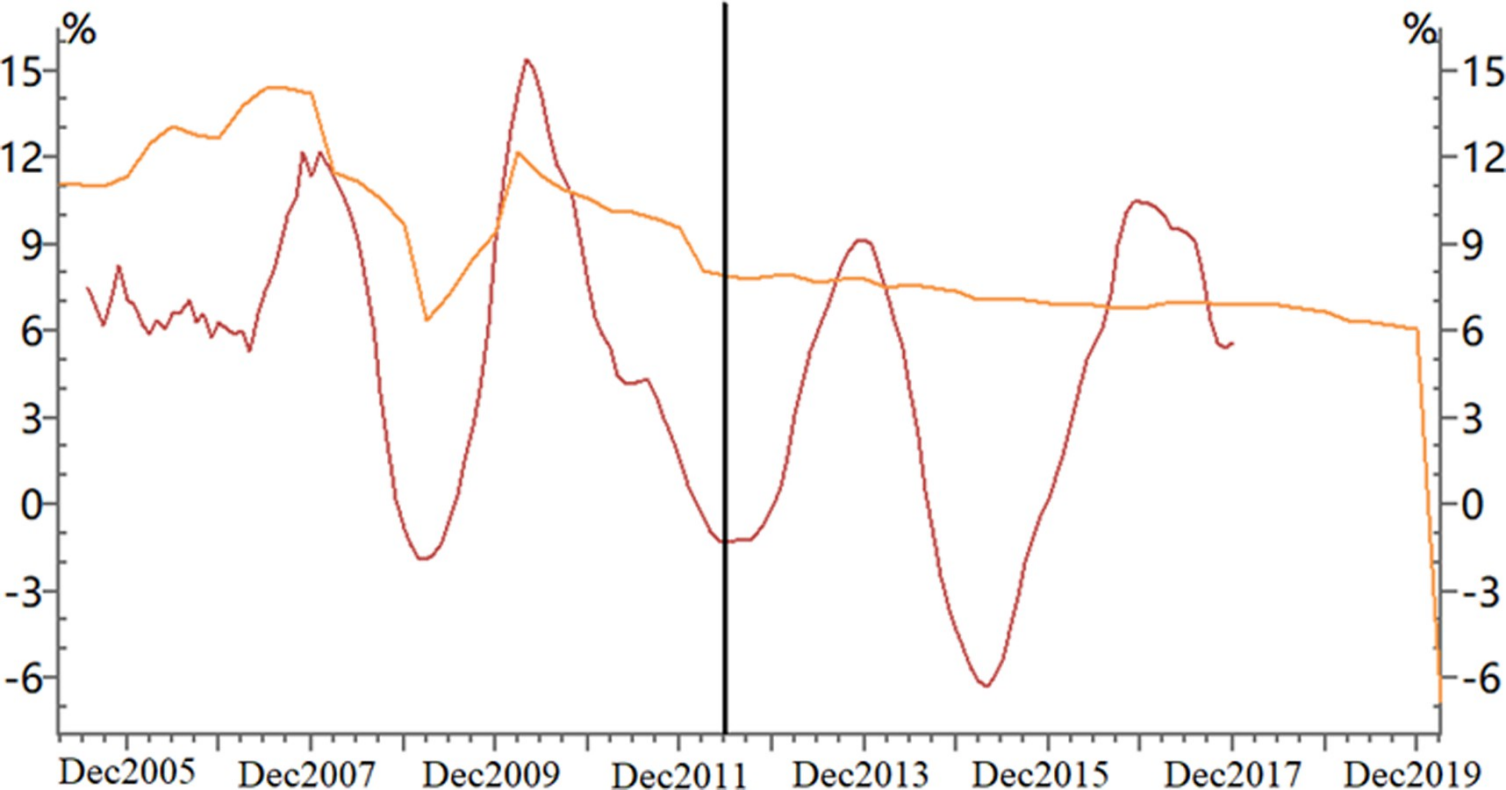
Growth rate of China’s economy and house prices. Among them: the orange line represents China’s real GDP growth, and the red line represents the price growth of new residential buildings in 70 major cities in China.

With the development of China’s industrialization and urbanization, China’s house prices are getting higher and higher. According to the data released by the China Statistical Bureau, house prices in Chinese cities have risen 4.8 times since 2000, and in some first-tier cities such as Beijing, Shanghai and Shenzhen, house prices have risen 7 to 9 times. According to data collected by Numbeo(www.numbeo.com), the annual ratio of house price to rent in China’s first-tier cities is 60–80, which is even much higher than that in the core cities of most developed countries. In the past, many scholars believed that the rise of house prices stimulates the rapid growth of the housing and relevant sectors, thus advance the economic growth. Yet in recent years, the relationship between house prices and China’s real economy growth has been gradually changing. Fig 1 shows that, from 2005 to 2012, China’s house prices are positive correlated with the real economy. But from 2012 to 2020, we find no obvious correlation between house price fluctuations and the real economy’s output, despite the significant rise of house prices during the years of 2012–2013 and 2015–2016. Neither of these two periods have the real economic output reacted much to the sharp rise of housing prices.

The accurate quantification of housing price bubbles and their implications for the macroeconomy form the core focus of this study. To this end, we employ a stochastic frontier model to compute the housing bubble index for China’s major cities and engage in empirical analysis to examine its specific effects on various macroeconomic indicators. Compared to previous studies, the innovations of this article include:(1) This study adopts the Stochastic Frontier Model to calculate the fundamental price of real estate, thereby estimating the housing bubble index for major Chinese cities. Compared to the traditional methods of using house price-to-income ratios and other indicators to gauge housing bubbles, our method offers a more accurate reflection of the relationship between house prices and the fundamental economy. This methodological approach represents a significant innovation in measuring housing bubbles, laying a solid foundation for subsequent research. (2) Based on the results of the housing bubble index calculation, we established a panel fixed-effects model using city-level data to empirically test the impact of housing bubbles on macroeconomic variables. Our findings contribute to a deeper understanding of the relationship between house prices and the macroeconomy, providing theoretical backing for macro controls in the real estate sector. (3) We further investigated whether the impact of housing bubbles on the macroeconomy varies across different economic development stages. Our results indicate that with shifts in the economic structure, the positive and negative effects of housing bubbles on the macroeconomy may reverse. This discovery enhances our understanding of the changes in China’s economic growth drivers and the necessity and urgency of structural adjustments.

As stated in the report issued at the 19th National Congress of the Chinese Communist Party, we never forget that housing is for living but not for speculation. Therefore, a housing system that ensures supply, housing purchase and renting brooks no delay. It is significant to investigate the effects of the over-rapid rise of housing prices on macroeconomic fluctuations, which would help construct a long-term regulatory mechanism of real estate, maintain financial stability, and achieve a long-term effective growth of the economy. In theory, our finding could not only enrich the theoretical model of housing and macroeconomy, but also help to evaluate the comprehensive influence of overhigh housing price on the macroeconomy, improve the efficacy of macro-economic regulation, and offer decision-making reference for the healthy development of real estate industry and real economy.

The remaining structure of this paper is organized as follows: The second section provides a literature review, the third section offers theoretical analysis and research hypotheses, the fourth section delves into the decomposition of real estate bubbles, the fifth section investigates the macroeconomic effects of housing bubbles, and the sixth section concludes the paper and presents relevant policy recommendations.

## 2. Literature review

### 2.1 Measurement of housing price bubbles

To clarify the relationship between the housing price bubble and the real economy, we need to measure the deviation of real estate prices relative to the real economy. Domestic and international literature on the measurement of housing price bubbles mainly divides into three categories:

Firstly, direct indicators such as house price-to-rent ratio, house price-to-income ratio, and vacancy rate are used as measures of relative real estate prices [1]. Although the house price-to-income ratio and the house price-to-rent ratio are straightforward, they might not accurately reflect the bubble level of China’s real estate prices. The main reasons are: (1) The house price-to-income ratio only considers income as a measure of the fundamental house price, neglecting the effects of population inflow and land supply on this fundamental price [2–4]. For instance, some economically developed cities in China attract a significant influx of population yet have limited land supply due to policy constraints [5]. Relying solely on the house price-to-income ratio to measure housing bubbles might underestimate the fundamental house price, resulting in an overestimation of the housing bubble.(2) The house price-to-rent ratio only considers rent as a determinant for the fundamental house price, overlooking other resources encapsulated within China’s real estate, such as household registration, education, and healthcare [6, 7]. Given that China’s residential rental market is predominantly short-term [8], current actual rents often reflect only the living value of real estate and hardly capture the value of resources like household registration and education that come with property ownership. Similarly, relying only on the house price-to-rent ratio might underestimate the fundamental house price, leading to an overestimation of the housing bubble.(3) Additionally, due to the inconsistency in statistical standards for residents’ income and real estate rent in China and the lack of a unified standard for selecting threshold values, both the house price-to-income ratio and house price-to-rent ratio struggle to accurately gauge the housing bubble index [3, 9].

Secondly, some scholars have used statistical tests to examine the growth rate of housing prices to determine whether a bubble exists in them. Diba and Grossman [10] identified asset price bubbles using unit root tests and cointegration tests. Phillips et al. [11] used right-tailed unit root tests to identify bubbles in the U.S. stock market. However, while statistical methods can detect asset price bubbles, they can’t explicitly calculate the fundamental value in asset prices, which might not align well with this study.

Lastly, some measure the fundamental value in asset prices using economic data. The primary approach is based on macroeconomic factors such as economic growth, resident income, construction costs, etc., to estimate the fundamental price of real estate, resulting in a housing price bubble index. For example, Gao et al. [12] built an expected equilibrium model, estimating the fundamental price of real estate based on basic factors influencing real estate prices. They found a certain housing price bubble in China’s major cities.

### 2.2 Macroeconomic effects

The impact of real estate prices on the macroeconomy has always been a focal point in academia. According to various theories and empirical studies, real estate prices show both stimulating and crowding-out effects on the macroeconomy.

From a stimulation perspective, there are two main aspects: firstly, the wealth effect triggered by rising house prices makes households feel wealthier, thus enhancing their willingness to consume and invest [13, 14]; secondly, the growth in house prices stimulates prosperity in the real estate and related industries, boosting government fiscal revenue and further supporting infrastructure investment [15, 16]. Both help expand the overall demand of the economy, promoting growth. From a crowding-out perspective, the main manifestations are: 1) High housing prices burden residents with heavier debt, decreasing their consumption capability [17–20]; 2) Real estate prosperity excessively crowds out investments in the real economy, even harming its innovation [21–24]. This phenomenon weakens the productivity and competitiveness of the real economy, resulting in a supply reduction. Moreover, some scholars believe that moderate housing price increases benefit the economy, but excessive growth might cause structural distortions in the economy, leading to crowding-out effects [15, 25]. Such distortions can disrupt resource allocation and market mechanisms, further affecting economic growth.

### 2.3 Literature review comments

While the achievements of past scholars are undeniable, there might still be certain limitations in their work: firstly, there lacks a unified and precise standard for measuring housing price bubbles. Many studies use indicators like the house price-to-income ratio to gauge housing bubbles, but these might overestimate the deviation of Chinese housing prices from economic fundamentals as they overlook other factors affecting house prices, such as population and land supply. Secondly, although many studies have explored the impact of housing bubbles on consumption, the economy, and exports, most of them use indicators like house price-to-income ratio to represent the housing bubble index. This simplification might introduce biases into bubble estimates, affecting the accuracy of studies on the relationship between housing bubbles and the macroeconomy. Lastly, there’s a lack of in-depth and detailed analysis of the mechanisms through which housing bubbles affect the macroeconomy. Many studies only focus on the overall effects of housing bubbles on consumption, investment, or output, without considering the heterogeneity of different economic development stages or city levels.

## 3. Theoretical analysis and research hypotheses

### 3.1 High house prices and consumption

Many studies find that high house prices could stimulate households’ consumption. Firstly, high house prices directly increase family wealth, which means that a family could gain additional income because the rising house prices induce the family to sell their house or moving to a smaller one. Meanwhile, the increase of real estate market-value also makes a family feel much richer and more willing to spend their money on consumption rather than saving or investment [13, 14]. Secondly, since the real estate can be used as mortgage, high housing price increases the market value of a house so that the family could obtain more low-interest loans through mortgaging their house to financial sector. The financial constraint is thus alleviated and the family can smooth their consumption [16, 26–28].

However, some studies found that the Chinese high house prices affect family’s consumption mainly through crowding-out effect, and they find no fortune effect. The reasons are as follows. Firstly, over-high house prices may lack wealth effect for those real estate holders. Some scholars have discovered that, when housing prices rise, most real estate holders cannot realize wealth-increase due to the constraints of rigid demands. Additionally, due to the imperfect development of the Chinese financial market and the limitations of real estate regulations, the Chinese housing loan market lacks second mortgage and refinancing. As a result, high house prices can’t realize its wealth effect in China [29–31]. Secondly, for those without a house or those seeking to improve their living conditions, higher house prices mean more decreased consumption payment and heavier housing loan. Hence, they have to minimize their current consumption, that is crowd-out effects. According to some studies, high house prices lead to residents’ precautionary savings, as they have to “save money for buying a house”. Apart from the growth of the household savings rate, the excessive rise of housing prices also leads to the drastic increase of resident debt and leverage in the household sector. In a word, high housing price crowds out residents’ level of consumption [18, 19, 29].

Based on the above arguments, we propose hypothesis H1: Housing price’s deviation from the basic price may decrease China’s resident level of consumption.

### 3.2 High housing price and macro-economy

Some studies believe that high house prices may be beneficial to the development of the real economy. For instance, some scholars believe that, in certain developing countries, the incomplete development of the financial market robs residents and enterprises of assets that could serve as collateral security and hedge. Since the real estate is an asset, high house prices relieve companies and families’ financial constraints, so that they can obtain more external financing, thus “squeeze into” the investments of real economy, promoting economic efficiency and growth [15, 16]. More and more scholars found high house prices’ crowding-out effect on the real economy in China. Their main reasons are as follows:

1. Effects of High House Prices on Real Economy Investment
  Over-high housing price brings a higher return rate of the real estate industry, which encourage more enterprises to invest in the real estate market and relevant industries. The increasing flow of financial factor flowing into the real estate sector creates the crowding-out effect on real economy’s investment. Some scholars have analysed the effects of high house prices on real economy’s investment from the perspective of investment efficiency. They found that the rising house prices attracts more foreign enterprises to the real estate market, along with massive funds for real estate investment, which stimulate the foreign investors’ demands for housing in the short run, and decrease the investment efficiency as well as total factor productivity, hence deteriorate resource allocation efficiency [21, 25, 32–34]. Some studies investigated the effects of high house prices on real economy’s investment from the perspective of credit resources’ allocation. They found that the “financial accelerator” effect brought by the high house prices encourage more credit resources to flow into the real estate sector through banks’ credit channels, thus crowding out the finance volume of the real economy. As the finance cost of the real economy increase, the efficiency of credit resources’ allocation is subsequently distorted [35, 36].
  Based on the above arguments, we propose hypothesis H2: House prices’ deviation from the fundamental value may crowd out China’s investment in the real economy.
2. Effects of High Housing Price on the Real Output
  Overhigh house prices not only crowd out investment from real economy, but also decrease the labour supply, innovation activities and total factor productivity. In short run, high house prices exert a crowding-out effect on the real economy. Some scholars found out that high housing price has increased the labour costs for the real enterprise and crowding out the labour supply [6, 22]. High house prices can affect the real economy through decrease firms’ R&D investments and individual initiative to start a business. Some studies found that cities with higher ratio of house price to income tend to have a lower possibility of entrepreneurship [23]. High house prices also can affect firms’ total factor productivity, and then induce the rapid growth of the real estate and relevant industries and the subsequent rise of economic growth rate. Meanwhile, rising house prices are also responsible for the rising costs of the real economy and the extrusion of other industries’ space for development. Furthermore, high housing price reduces the industrial enterprises’ margin revenue and the total factor productivity and is therefore not conducive to the development of the real economy [37–39].
  Based on the above arguments, we propose hypothesis H3: House prices’ deviation from the basic price may crowd out the level of the Chinese real economy’s output.

## 4. Housing price bubble measurement

This section mainly introduces the way to evaluate the fundamental value of house prices. First, we construct the stochastic frontier model and calculate the fundamental value. Second, the index representing the degree of housing price’s deviation from the fundamental value is calculated.

### 4.1 Theoretical model

The literature review discussed methods suggesting that we can estimate the fundamental value in the asset price using economic data. In this paper, we adopt the stochastic frontier model to estimate the basic housing value base on the finding of Battese [40, 41] and Greene [42]. The stochastic frontier model derives from the estimation of the production function and the cost function in economics. Take estimation of the production function as an example. All enterprises in the hypothetical economy are faced with the cost function, formally known as the Cobb-Douglas function. But corporate costs often fail to meet the standard hypothesis. Therefore, the stochastic frontier model assumes that there exists an inefficient item, which represents the difference between firm’s real productivity and its optimized efficiency. Luo et al. [43] and Yi et al. [44] estimate the bubble index of the stock price with the stochastic frontier model in which they construct the production function between stock value and estimated value.

Base on the studies of Bernanke et al. [45] and Martin et al. [25], we divide the house prices *Q*_*t*_ observed in reality into three parts: the fundamental value $Q_{t}^{b}$ which represents the residential feature of housing, the investment price $Q_{t}^{iv}$ which represents investment value of housing, and the random shock *ξ*_*t*_ which affects house prices. *ξ*_*t*_ satisfies normal distribution $\xi_{t}\in N\left(0,\sigma_{\xi }^{2}\right)$, and σ_ξ_ is the standard deviation of external house price fluctuations. It can be illustrated as follow.

$$Q_{t}=Q_{t}^{b}+Q_{t}^{iv}+\xi_{t}=F_{b}\left(x_{1}^{b},x_{2}^{b},x_{3}^{b}\ldots \right)+F_{iv}\left(x_{1}^{iv},x_{2}^{iv},x_{3}^{iv}\ldots \right)+\xi_{t},\left(\xi_{t}\in N\left(0,\sigma_{\xi }^{2}\right)\right)$$ (1)

$$Q_{t}^{b}=F_{b}\left(x_{1}^{b},x_{2}^{b},x_{3}^{b}\ldots \right)$$ (2)

$$Q_{t}^{b}=F_{b}\left(x_{1}^{b},x_{2}^{b},x_{3}^{b}\ldots \right)$$ (3)

$$Q_{t}^{iv}=F_{iv}\left(x_{1}^{iv},x_{2}^{iv},x_{3}^{iv}\ldots \right)$$ (4)

where $x_{i}^{b}$represents the factors affecting the fundamental value of housing like citizens’ income level, growth of urban population, and urban land supply. These factors represent the fundamental factors that affect the house prices, and through changing people’s demands for housing. $x_{j}^{iv}$represents the factors which could affect housing’s investment price, such as the degree of asset shortage, the money supply changes, real estate credit rate and the percentage of the down payment for real estate loans. These factors affect housing prices through shifting citizens investment demands or speculation demands.

We assume the housing bubble index *k*_*t*_. When *k*_*t*_ = 1, the housing price totally reflects its fundamental value. We introduce the housing bubble index *k*_*t*_ into the formula(1) and obtain the following formula:

$$Q_{t}=Q_{t}^{b}\kappa_{t}e^{\xi_{t}},\left(\xi_{t}\in N\left(0,\sigma_{\xi_{t}}^{2}\right)\right)$$ (5)

where we define the fundamental housing value $Q_{t}^{b}=F_{b}\left(x_{i}^{b},\beta \right)$. $x_{i}^{b}$is the factor affecting fundamental value, *β* are the estimated parameter for the corresponding variables. Formula (5)can be rewritten as:

$$Q_{t}=F_{b}\left(x_{i}^{b},\beta \right)\kappa_{t}e^{\xi_{t}},\left(\xi_{t}\in N\left(0,\sigma_{\xi_{t}}^{2}\right)\right)$$ (6)

Suppose $F_{b}\left(x_{i}^{b},\beta \right)=e^{\beta_{0}^{b}}\prod_{i=1}^{k}x_{1t}^{\beta_{i}^{b}}$, where *k* represents the number of factors affecting fundamental housing value, We take the logarithm from both sides of formula(5), and order that *μ*_*t*_ = In *k*_*t*_. Then we obtain:

$$\text{ln}Q_{t}=\beta_{0}^{b}+\sum_{i=1}^{m}\beta_{i}^{b}\text{ln}x_{it}^{b}+\mu_{t}+\xi_{t}$$ (7)

To estimate *u*_*t*_, some assumptions about the distribution of the housing bubble index *u*_*t*_ are needed. The assumptions are as followed:

1. The housing price is no smaller than its fundamental price, which means the housing bubble index *μ*_*t*_ ≥ 1.
2. The housing bubble index *μ*_*t*_ is independent of the residual item *ξ*_*t*_, and both are iid. Assume that *ξ*_*t*_ befits normal distribution, then $\xi_{t}\in N\left(0,\sigma_{\xi_{t}}^{2}\right)$. Since *μ*_*t*_ ≥ 1, we assume that *μ*_*t*_ is consistent with half-normal distribution, $\xi_{t}\in N\left(0,\sigma_{\xi_{t}}^{2}\right)$. *ξ*_*t*_ and *μ*_*t*_ are independent of each other, and their density of simultaneous distribution is as formula(8). σ_*μ*_ and σ_*ξ*_ are, respectively, standard deviations of the corresponding variables.
$$f\left(u_{i},\xi_{i}\right)=\frac{1}{\pi \sigma_{\mu }\sigma_{\xi }}\exp\left\{-\frac{u_{i}^{2}}{2\sigma_{\mu }^{2}}-\frac{\xi_{i}^{2}}{2\sigma_{\xi }^{2}}\right\}$$ (8)
3. With unchanging macroeconomic conditions, the degree of housing price’s deviation from its fundamental value shrinks to 1 along with the passage of time. We use *g*(*t*) to represent the shrinkage of the housing bubble along with time, illustrated in formula(9). It means that, without exogenous shocks, the house price bubble would gradually vanish along with time. Here, *η* denotes the convergence coefficient of the housing bubble, T denotes the temporal dimension. This hypothesis satisfies the self-correlation in the degree of housing price’s deviation.

$$g(t)=e^{-\eta (t-T)}$$ (9)

Based on the above model and hypothesis, we draw on the stochastic frontier model constructed by Battese [40, 41] and Greene [42], with which we may estimate the logarithm value of housing price’s degree of deviation from the basic price *μ*_*t*_.

### Variables and data specifications

1. Fundamental housing value. Real estate is both a consumer product and an item for investment. The fundamental housing value should reflect both its attribute as consumer goods and the condition of the macro-economic fundamentals. This paper adopts the following variables to assess the fundamental housing value. (a) The levels of resident income and population. Both resident income and population affect the demand for housing. Since the Reform and Opening-up, the ever-rising Chinese resident income and level of urbanization are two key factors contributing to the escalating house prices [46–49]. (b) Land supply. Land supply affects the supply of the housing market. If the growth rate of land supply fails to meet the increasing demands, it may lead to excessive growth of the housing prices [5, 50–52].
2. Data Specifications. The samples in this paper come from panel data of 35 large and medium-sized cities in China from 1999 to 2019(In order to avoid the impact of the COVID-19 Pandemic on the conclusions of this analysis, we use the data before the outbreak of the epidemic for empirical testing). Here, the variables adopted for assessing the housing bubble include price level, resident income, household population, the average wage of staff and land supply. Apart from the housing bubble index which is obtained via assessment, all the other basic data come from official statistics, including the Wind Economic Database, website of the People’s Bank of China, and National Bureau of Statistics website. The descriptive statistics for the data are presented in Table 1.

**Table 1:** Descriptive statistics.

Variables	Sample Size	Mean	Standard Deviation	Min	Max
House Price	607	6747	6009	1077	55441
Household Population	620	560.1	268.3	54.38	1476
Land Supply Per Capita	292	25.85	6.664	13.10	50.11
GDP Deflator	700	111.8	3.461	94.20	130.9
Average Wage	680	40124	25722	7408	149843
City Dummy Variables	770	0.11489	0.31912	0	1
City Number	770	18	10.106	1	35

### 4.3 Empirical results analysis

#### 4.3.1 Analysis of the stochastic frontier model’s results

The results are illustrated in Table 2. In the initial model, we included two sets of key control variables: individual fixed effects, which help control potential city-specific effects, and time-fixed effects aiming to capture generic influences at specific time points, such as the 2007 financial crisis. In Model I, the independent variable includes average wages. Model II incorporates urban household population as an additional independent variable. In Model III, the land supply is added as an independent variable. Wherein, Sigma_u represents the variance of the efficiency error term; Sigma_v is the variance of the random error term; λ indicates the ratio of the standard deviation of efficiency error to random error. A larger λ means that the efficiency error plays a dominant role in the total error, suggesting that the setting of the Stochastic Frontier Model is highly effective.

**Table 2:** Analysis of the stochastic frontier model’s results.

	Model I	Model II	Model III	Model IV	Model V
Variables	Housing Price	Housing Price	Housing Price	Housing Price	Housing Price
Average Wages	0.915***	0.841***	0.845***	1.060***	1.071***
	(0.0215)	(0.0159)	(0.0160)	(0.0656)	(0.0756)
Household Population		0.185***	0.168***	0.199***	0.143***
		(0.0159)	(0.0168)	(0.0234)	(0.0393)
Land Supply			-0.463***	-0.317***	-0.354***
			(0.0434)	(0.060)	(0.0396)
Time Trend				0.0777***	0.0205**
				(0.00764)	(0.00886)
City Dummy Variables					0.537***
					(0.0481)
Individual Effect	YES	YES	YES	YES	YES
Time Effect	YES	YES	YES	YES	YES
Intercept term	-1.362***	-1.769***	-1.698***	-2.898***	-3.127***
	(0.222)	(0.185)	(0.187)	(0.422)	(0.384)
Sigma_u	2.057	4.041	3.489	3.274***	3.119***
	(3.655)	(3.132)	(3.438)	(0.117)	(0.116)
Sigma_v	0.190***	0.115***	0.126***	0.0938	0.284
	(0.0141)	(0.0095)	(0.00984)	(0.0965)	(0.434)
λ	10.783***	34.986***	27.634***	5.004	5.483**
	(3.651)	(3.132)	(3.438)	(2.316)	(2.595)
Observations	587	525	525	238	240
ID	35	35	35	31	31

Standard errors in parentheses. And

*** p<0.01

** p<0.05

* p<0.1.

From Table 2 we can draw the following conclusions. Firstly, the average wages, household population and land supply all significantly affect the house prices. The average wage level of residents is one of the important factors affecting real estate demand. The rise in the average wage of residents reflects the growth of residents’ income. Income growth will encourage residents to buy or replace better housing to improve their quality of life [19]. Empirical analysis shows that the growth of the average wage of residents is positively correlated with real estate prices, which is consistent with economic theory expectations. As a supply-side factor in the housing market, land supply increases are negatively correlated with the house prices, which is mostly consistent with the economic expectations. Secondly, *λ*, the ratio of housing price’s deviation level to its random error, is much larger than 1, which indicates the dominating position assumed by the housing bubble level. Thus, the stochastic frontier model can effectively dissociate the fundamental price from the house prices.

To further ensure the robustness of our regression results, we introduced the following control variables into the model: Firstly, we incorporated a time trend term to capture potential time trends within the data, as shown in Model IV; Secondly, we also added city dummy variables to further control for specific effects of individual cities, as illustrated in Model V. It’s worth noting that even after considering these additional control variables, our core findings remain consistent with the baseline model, further confirming the robustness of our research conclusions.

#### 4.3.2 Analysis of urban house price deviation

**Fig 2:**
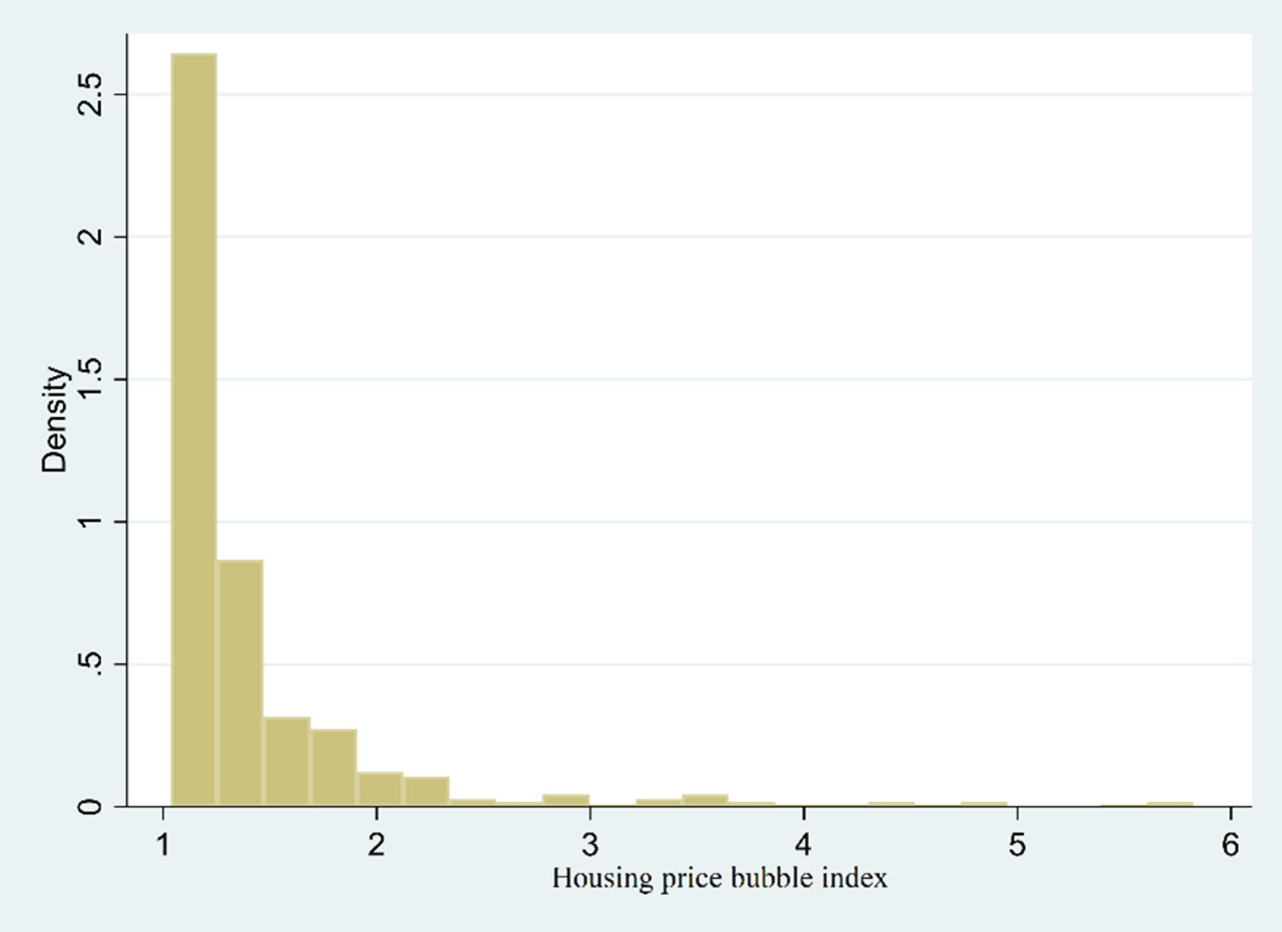
The probability density function of the degree of the housing price bubble.

Based on the analysis of the above model, having obtained the bubble value *u*_*t*_ of each city at a given time, we then compute the degree of housing price’s deviation as $Q/Q_{t}^{b}=e^{u_{t}}$. Table 3 provides the descriptive statistics of the housing bubble index. Table 3 reveals that China’s average housing bubble index is 1.44, and the averaged housing bubble index in first-tier cities reaches as high as 2.70. Fig 2 reports the probability density function of housing price bubble indexes. In Fig 2, we can see that $Q/Q_{t}^{b}$ remains within the section [1, 2] in most samples, which indicats that the degree of the housing bubble remains in most Chinese second-and-third tier cities. For $Q/Q_{t}^{b}$, only a few $Q/Q_{t}^{b}$ are within [2,+∞), which demonstrates the housing bubble level of China’s first-tier cities. The above distinction reveals the divergence of housing price’s deviation from the fundamental value in the first-tier and the second-and-third tiers cities respectively. These results are consistent with many existing studies. For instance, Meng et al. [53] discovered that the disparate distribution of income, welfare, and infrastructure across various urban regions has led to cross-regional population migration, subsequently elevating housing prices in China’s first-tier cities. Han and Lu [5], along with Wang and Richman [53] highlighted that constrained land supply in first-tier cities, paired with robust residential demand, has fostered a persistent divergence in housing prices between China’s first-tier cities and smaller, medium-sized cities.

**Table 3:** Descriptive statistics of housing bubble index.

Variables	Average Value	Standard Deviation	Minimum Value	Maximum Value	Observed Value
Housing price bubble	1.44	0.66	1.04	5.82	525
First-tier cities’ housing price bubble	2.7	1.37	1.21	5.82	34

## 5. Macro-economic effects of the high house prices

In this section, we empirically investigate the impacts of high house prices on the macro-economy with the index for the degree of housing price’s deviation from the basic price (measured in the first section) and macro-economic data of China’s cities.

### 5.1 Empirical strategyand data sources

To test the hypotheses H1, H2 and H3 proposed earlier, based on the studies of Rong and Wang [54], Zhang et al. [1], and Chen and Zhang [55], we construct a fixed-effects model with urban panel data. The empirical model adopted for benchmark return is as follows:

$${\left[{\text{C}}_{it},inv_{it}^{\gamma },\alpha inv_{it}^{h},\gamma 1_{\text{it}},\gamma_{\text{it}}\right]}^{\text{T}}=\kappa +\alpha u_{\text{t}}+\gamma X_{it}+\mu_{t}+v_{t}+\varepsilon_{it}$$ (10)

#### 5.1.1 Dependant variable

Covering the three aspects of consumption, investment and output. The set of dependant variables includes the following five variables: (1) C_*it*_ denotes household consumption of city *i* at *t* period; (2) $inv_{it}^{r}$ is the real economic investment of city *i* at *t* period; (3) $\alpha inv_{it}^{h}$ presents the proportion of real estate investment in the fixed assets of city *i* at *t* period; (4) $\gamma 1_{\text{it}}$ denotes the real economic output of city *i* at *t* period; (5) $\gamma_{\text{it}}$ is the GDP output *u*_*t*_ of city *i* at *t* period.

#### 5.1.2 Independent variable

The index presenting house prices’ deviation from the fundamental value for city *i* at *t* period is obtained from the model built in this section, and it reflects the degree of housing price’s deviation from the economic fundamentals.

#### 5.1.3 Control variables

X_it_ denotes the control variable, including other variables that affect output and consumption. It includes the following aspects: (1) individual effects in the city *μ*_*i*_; (2) time fixed effects *v*_*t*_; (3) time trend; (4) other variables proxied urban features, like per capita living space, total fixed asset investment, total imports and exports, revenue within the regional fiscal budget, educational level, post and telecommunication business, and highway network coverage rate.

We have collected key economic indicators from 35 major cities in China to deeply analyze the impact of the housing bubble on the macroeconomy. Specifically, (1) the real estate price bubble index *u*_*t*_ is calculated from the stochastic frontier model presented earlier in this paper; (2) residents’ consumption C*_it_* is proxied by the per capita consumption expenditure of urban residents; (3) real economic investment $inv_{it}^{r}$ is estimated by subtracting real estate investment from total fixed asset investment; (4) the proportion of real estate investment to fixed assets $\alpha inv_{it}^{h}$ is derived from the ratio of real estate investment to total fixed asset investment; (5) real economic output *Y*1_it_ is reflected using the total industrial output value of the city. To mitigate potential impacts on the analysis due to the COVID-19 Pandemic, our chosen sample period is from 2003 to 2019, explicitly avoiding the outbreak period. All data used in this study were sourced from the Wind Financial Database, the official website of the People’s Bank of China, and the official website of the National Bureau of Statistics, ensuring the accuracy and reliability of the data. The descriptive statistics for the data are presented in Table 4.

**Table 4:** Descriptive statistics.

Variables	Sample Size	Mean	Standard Deviation	Min	Max
Housing Price Bubble Index	587	1.381	0.455	1.055	4.563
Real Economic Output	558	4165	4932	66.12	32119
Per Capita Consumer Expenditure	569	16789	8645	4789	46015
Local Fiscal Revenue	620	3.34e+06	4.212e+06	43767	3.540e+07
Real Economic Investment	654	2.26e+07	2.480e+07	396484	1.750e+08
Per Capita Housing Area	292	25.85	6.664	13.10	50.11
Total Import and Export Value	449	3.245e+06	6.678e+06	36877	5.370e+07
Real Estate Investment	650	797.0	885.7	10.51	4439

### 5.2 Main results

#### 5.2.1 Results for household consumption

Table 5 reports the empirical results of the effects of house prices’ deviation from the fundamental price on household consumption. Column 4 shows that a 1% increase of house prices’ deviation from the basic price household consumption is associated with 0.07% lower consumption. After restructuring the controlled variable, the household consumption level would drop by 0.11% at maximum. It indicates that, the larger the deviation of housing price from the economic fundamentals, the greater the crowing effect on household consumption level. The greater the deviation of housing price from the economic fundamentals, the more the expenditure spent by residents for real estate purchase. Given the fact that most families purchase housing with loans, high housing price crowds out household consumption. This result is consistent with conclusions drawn by Yan and Zhu [29] and Pan and Liu [19]. Thus we do not reject the hypothesis H1.

**Table 5:** Impacts of housing price’s deviation from the basic price on consumption.

Variables	Household consumption	Household consumption	Household consumption	Household consumption
Housing price bubble index	-0.102**	-0.113**	-0.113**	-0.0727**
	(0.0424)	(0.0435)	(0.0435)	(0.0331)
Per capita living space		0.0332	0.0332	0.0362
		(0.0729)	(0.0729)	(0.0692)
Time trend			0.0807***	0.0562***
			(0.00329)	(0.00879)
Total amount of fixed-asset investment				0.137**
				(0.0527)
Intercept term	8.969***	8.936***	-152.6***	-105.6***
	(0.0677)	(0.245)	(6.357)	(16.88)
Controlled Variables	YES	YES	YES	YES
Temporal effects	YES	YES	YES	YES
Individual effects	YES	YES	YES	YES
Observations	496	237	237	236
R-squared	0.968	0.957	0.957	0.96
Number of id	31	31	31	31

#### 5.2.2 Results for investment

Table 6 is the empirical results of the impacts of housing price’s deviation from the basic price on real economy’s level of investment. The results reveal that every time the housing price’s deviation from the basic price increases by 1%, the real economy’s level of investment would drop by 0.169%. It indicates that, the larger the deviation of housing price from the economic fundamentals, the greater the crowing effect on real economy’s investment. To further verify the negative impacts of housing price’s deviation from the basic price on real economy’s level of investment, we examine the impacts of housing price’s deviation from the fundamental value on real estate investment proportion. The results are listed in Table 7. The results reveal that every time the housing price’s deviation from the basic price increases by 1%, the real economy’s level of investment would drop by 0.151%. It means that the higher the housing price, the larger proportion of real estate investment in fixed-asset investment, and the smaller the proportion of real economic investment. Thus we do not reject the hypothesis H2.

**Table 6:** Impacts of housing price’s deviation from the basic price on real economic investment.

Dependent Variables	Real economic investment			
Housing price bubble index	-0.228***	-0.154**	-0.154**	-0.169***
	(0.0504)	(0.0577)	(0.0577)	(0.0561)
Total value of imports and exports		0.126*	0.126*	0.00998
		(0.0651)	(0.0651)	(0.0704)
Time trend			0.194***	0.0534
			(0.0130)	(0.0520)
Regional revenue within fiscal budget				0.905***
				(0.296)
Intercept term	14.74***	12.62***	-376.0***	-104.6
	(0.0798)	(0.829)	(25.28)	(100.4)
Controlled Variables	YES	YES	YES	YES
Temporal effects	YES	YES	YES	YES
Individual effects	YES	YES	YES	YES
R-squared	0.909	0.876	0.876	0.898
Clustering number	31	31	31	31

**Table 7:** Impacts of housing price’s deviation from the basic price on real economic investment.

Variables	Proportion of real estate investment to fixed investment			
Housing price bubble index	0.176***	0.133***	0.151***	0.151***
	(0.0219)	(0.0273)	(0.0288)	(0.0288)
Total value of imports and exports		0.0300**	-0.0115	-0.0115
		(0.0126)	(0.0246)	(0.0246)
Regional revenue within fiscal budget			0.0755*	0.0755*
			(0.0385)	(0.0385)
Time trend				-0.00136
				(0.0205)
Controlled Variables	YES	YES	YES	YES
Temporal effects	YES	YES	YES	YES
Individual effects	YES	YES	YES	YES
Constant	-10.85***	-11.26***	11.78***	-9.067
	(0.0669)	(0.318)	(0.414)	(41.25)
Observations	494	431	431	431
R-squared	0.164	0.183	0.19	0.19
Clustering number	31	31	31	31

#### 5.2.3. Effects on output

Table 8 presents the empirical results of the impact of housing bubbles on GDP and output of the real economy. Firstly, moving from left to right, we sequentially add different control variables to the regression model. The results show that the regression coefficient of the core variable, the housing bubble index, remains relatively stable, suggesting that the regression outcomes are robust. Secondly, the results indicate that the regression coefficient of the core variable is negative and is significant both statistically and economically. Specifically, with a 1% increase in the housing bubble index, the total economic output will decrease by 0.07%, and the output of the real economy will decline by 0.209%. This suggests that the higher the level of the housing bubble, the greater the crowding-out effect on GDP and the output of the real economy. Thus we do not reject the hypothesis H3.

**Table 8:** Impacts of housing price’s deviation from the basic price on economic growth.

Variables	GDP	Real economy	GDP	Real economy	GDP	Real economy	GDP	Real economy
Housing price bubble index	-0.0462*	-0.236***	-0.120***	-0.334***	-0.120***	-0.334***	-0.070***	-0.209***
	(0.0241)	(0.0676)	(0.0282)	(0.0602)	(0.0282)	(0.0602)	(0.0250)	(0.0605)
Per capita living space			0.0979*	0.163	0.0979*	0.163	0.102*	0.172
			(0.0564)	(0.151)	(0.0564)	(0.151)	(0.0529)	(0.138)
Time trend					0.140***	0.195***	0.112***	0.0975***
					(0.00264)	(0.0111)	(0.00974)	(0.0238)
Fixed asset investment							0.169***	0.428***
							(0.0557)	(0.117)
Intercept term	15.94***	6.826***	15.81***	6.537***	-263.6***	-383.9***	-210.9***	-195.2***
	(0.0274)	(0.0868)	(0.174)	(0.456)	(5.113)	(21.85)	(18.69)	(45.97)
Controlled Variables	YES	YES	YES	YES	YES	YES	YES	YES
Temporal effects	YES	YES	YES	YES	YES	YES	YES	YES
Individual effects	YES	YES	YES	YES	YES	YES	YES	YES
Observations	525	463	238	236	238	236	237	236
R-squared	0.977	0.932	0.982	0.928	0.982	0.928	0.984	0.941
Number of id	31	31	31	31	31	31	31	31

### 5.3 Analysis of heterogeneity

#### 5.3.1 Effects of temporal classification

Following the financial crisis of 2008, the Chinese government implemented an economic stimulus package worth four trillion yuan, leading to rapid growth in real estate investments and soaring housing prices. Subsequently, between 2010 and 2011, to stabilize the real estate market, China introduced a series of stringent regulatory measures, such as the "National Ten Articles". These policies included raising down payments, interest rates, and taxes, as well as imposing restrictions on purchasing a second home and on non-local residents buying properties. The loose monetary policy had fuelled over-investment in real estate. However, the subsequent tight regulations resulted in a scenario where high housing inventory coexisted with rising housing prices. Data from the Shanghai E-House Real Estate Research Institute indicates that in 2012 and 2014, the property inventory-to-sales ratio stood at a staggering 22.8 months and 20.9 months, respectively. Hence, 2011 stands as a significant turning point in China’s real estate regulatory landscape, which is why we chose 2011 as the pivotal year.

Tables 9 and 10 are the empirical results of the impacts of housing price’s deviation from the basic price on GDP and real economy’s output in respective groups of the temporal dimension. The results lead to the following revelations. Firstly, at a different stage of China’s economic development, housing prices’ deviation from the basic price holds different impacts on the macroeconomy. Along with China’s economic growth, the impacts left housing price’s deviation from the basic price on GDP and the real economy gradually changed from evidently positive to evidently negative. Secondly, along with China’s economic growth, the crowding-out effect exerted by housing price’s deviation from the fundamental value on the real economy should be much larger than that exerted on the GDP. Tables 11 and 12 report the regression results of the effects of housing price’s deviation from the basic price on real economic investment and household consumption in respective groups of the temporal dimension. The results are as follows. Firstly, along with China’s economic growth, the impacts left by housing price’s deviation from the basic price on the growth of real economic investment gradually changed from evidently positive to evidently negative. Secondly, throughout the entire temporal dimension, the impact on consumption left by housing price’s deviation from the basic price remains negative, and it becomes more evident along with time.

**Table 9:** Output responses to changes in house prices’ deviation from the fundamental prices.

Variables	GDP		Substantial economic output	
Years	2003–2011	2012–2019	2003–2011	2012–2019
Housing price bubble index	0.0396**	-0.0994***	0.0630**	-0.311***
	(0.0172)	(0.0249)	(0.0266)	(0.0622)
Intercept term	17.43***	16.01***	8.436***	6.907***
	(0.0193)	(0.0245)	(0.0290)	(0.0798)
Controlled Variables	YES	YES	YES	YES
Time trend	YES	YES	YES	YES
Temporal effects	YES	YES	YES	YES
Individual effects	YES	YES	YES	YES
Observed Value	217	308	155	308
R-squared	0.846	0.979	0.247	0.953
Clustering number	31	31	31	31

**Table 10:** Effects of house prices’ deviation from the fundamental prices on output.

Variables	GDP				Substantial economic output			
Years	2003–2006	2007–2010	2011–2014	2015–2019	2003–2006	2007–2010	2011–2014	2015–2019
Housing price bubble index	0.0423	0.0989***	-0.029	-0.0614**	0.240*	0.202***	-0.111	-0.117*
	(0.0421)	(0.0243)	(0.0350)	(0.0292)	(0.121)	(0.0714)	(0.0690)	(0.0600)
Intercept term	15.39***	15.95***	16.42***	16.77***	7.030***	7.997***	8.226***	8.574***
	(0.0498)	(0.0314)	(0.0525)	(0.0329)	(0.142)	(0.0940)	(0.104)	(0.0780)
Controlled Variables	YES	YES	YES	YES	YES	YES	YES	YES
Time trend	YES	YES	YES	YES	YES	YES	YES	YES
Temporal effects	YES	YES	YES	YES	YES	YES	YES	YES
Individual effects	YES	YES	YES	YES	YES	YES	YES	YES
Observed Value	136	138	140	105	124	122	124	62
R-squared	0.964	0.961	0.938	0.691	0.935	0.841	0.734	0.06
Clustering number	34	35	35	35	31	31	31	31

**Table 11:** Effects of house price’ deviation from the fundamental price on investment and consumption.

Variables	Substantial economic investment		Consumption:	
Years	2003–2011	2012–2019	2003–2011	2012–2019
Housing price bubble index	0.281***	-0.119*	-0.0177	-0.0357*
	(0.0632)	(0.0657)	(0.0495)	(0.0181)
Intercept term	14.81***	16.55***	15.12***	16.65***
	(0.0791)	(0.0706)	(0.105)	(0.0286)
Controlled Variables	YES	YES	YES	YES
Time trend	YES	YES	YES	YES
Temporal effects	YES	YES	YES	YES
Individual effects	YES	YES	YES	YES
Observed Value	187	307	217	258
R-squared	0.226	0.922	0.961	0.963
Clustering number	31	31	31	31

**Table 12:** Impacts of housing price’s deviation from the basic price on investment and consumption.

Variables	Substantial economic investment				Consumption:			
Years	2003–2006	2007–2010	2011–2014	2015–2019	2003–2006	2007–2010	2011–2014	2015–2019
Housing price bubble index	0.247*	0.0958	-0.289**	0.101	0.001	-0.041	0.045***	-0.202
	(0.132)	(0.142)	(0.115)	(0.223)	(0.023)	(0.030)	(0.016)	(0.155)
Intercept term	16.83***	16.49***	16.31***	14.66***	16.04***	15.61***	14.90***	14.46***
	(0.152)	(0.212)	(0.159)	(0.274)	(0.029)	(0.045)	(0.022)	(0.183)
Controlled Variables	YES	YES	YES	YES	YES	YES	YES	YES
Time trend	YES	YES	YES	YES	YES	YES	YES	YES
Temporal effects	YES	YES	YES	YES	YES	YES	YES	YES
Individual effects	YES	YES	YES	YES	YES	YES	YES	YES
Observed Value	133	137	140	105	109	138	140	105
R-squared	0.869	0.867	0.765	0.022	0.857	0.992	0.978	0.87
Clustering number	34	35	35	35	34	35	35	35

The above conclusions indicate that the promoting effect and crowding out effect on macroeconomy caused by housing price’s deviation from the fundamental value counterbalance against each other. In the early stage of China’s economic development, the rising housing price can quickly promote China’s economic growth through expansion of the real estate industry and promotion of relevant industries. Along with Chinese urbanization and the drastic uplift of the aggregate economic volume, the promoting effect brought by the real estate industry gradually diminishes and the crowding-out effect begins to take over. As a result, the high house prices gradually create more negative impacts on China’s macro-economy. The impact of the real estate market on the real economy has both direct effects, such as promoting the growth of the real estate and related industries, and indirect effects, such as stimulating economic growth through the wealth effect. However, a plethora of empirical studies indicates that the indirect effects resulting from rising real estate prices in China aren’t very pronounced and sometimes even have adverse impacts [17–19]. Additionally, the high inventory in the real estate market suggests that the direct effects of housing price bubbles on the real economy are not very evident. This might explain why, post-2011, there was an inverse relationship between housing price bubbles and the real economy.

#### 5.3.2. Urban grouping effect

According to the classification by Ni [56], we categorize the 35 cities into first-tier, second-tier, and third-tier cities. The first-tier cities comprise Beijing, Shanghai, Shenzhen, and Guangzhou. Second-tier cities include provincial capitals (except the first-tier cities), sub-provincial cities, and those designated in the state plan. Third-tier cities refer to those not included in the first two tiers.

Table 13 presents the regression results of the impact of housing price deviations from their fundamental value on GDP and the real economy’s output across different urban dimensions. Two primary observations emerge. First, in first-tier cities, deviations in housing prices from the fundamental value may exert some positive influence on GDP and the real economic output. However, this positive effect is statistically marginal. A 1% increase in the deviation leads to a mere 0.026% rise in the GDP of first-tier cities. Second, housing price deviations have a crowding-out effect on GDP and real economic output in both second-tier and, more pronouncedly, third-tier cities.

**Table 13:** Impacts of house prices’ deviation from the base price on real economic investment.

	GDP			Real economy		
Years	First-tier	Second-tier	Third-tier	First-tier	Second-tier	Third-tier
Housing price bubble index	0.0258**	-0.0353	-0.210**	0.112***	-0.272***	-0.752***
	(0.00920)	(0.0299)	(0.0706)	(0.0162)	(0.0662)	(0.157)
Intercept term	17.20***	16.11***	15.18***	8.113***	7.077***	6.288***
	(0.0245)	(0.0307)	(0.102)	(0.0767)	(0.0595)	(0.223)
Controlled Variables	YES	YES	YES	YES	YES	YES
Time trend	YES	YES	YES	YES	YES	YES
Temporal effects	YES	YES	YES	YES	YES	YES
Individual effects	YES	YES	YES	YES	YES	YES
Observed Value	34	373	118	32	329	102
R-squared	0.999	0.974	0.988	0.985	0.934	0.939
Clustering number	4	22	7	4	22	7

Table 14 showcases the regression results concerning the effects of housing price deviations from the fundamental value on real economic investment and household consumption across urban dimensions. The findings suggest that, initially, such deviations don’t markedly influence real economic investment and consumption. Secondly, these deviations display a pronounced crowding-out effect on real economic investment and consumption in both second-tier and, especially, third-tier cities.

**Table 14:** Impacts of housing price’s deviation from the basic price on real economic investment.

	Substantial economic investment			Consumption:		
	First-tier	Second-tier	Third-tier	First-tier	Second-tier	Third-tier
Housing price bubble index	0.0188	-0.178**	-0.388**	0.0162	-0.0682***	-0.0445*
	(0.0390)	(0.0767)	(0.143)	(0.0300)	(0.0208)	(0.0306)
Intercept term	15.41***	14.80***	14.26***	16.06***	15.19***	15.03***
	(0.0983)	(0.0987)	(0.109)	(0.0798)	(0.0604)	(0.0417)
Controlled Variables	YES	YES	YES	YES	YES	YES
Time trend	YES	YES	YES	YES	YES	YES
Temporal effects	YES	YES	YES	YES	YES	YES
Individual effects	YES	YES	YES	YES	YES	YES
Observed Value	32	352	110	34	339	102
R-squared	0.985	0.908	0.969	0.993	0.989	0.994
Clustering number	4	22	7	4	22	7

Empirical regression results highlight that while deviations in housing prices from their fundamental value have certain positive effects in first-tier cities, they don’t justify the exceptionally high housing prices there. Given their robust economy and remarkable per capita GDP, Chinese first-tier cities possess a solid economic foundation supporting their elevated housing prices. However, these high prices intensify the residents’ real estate purchasing burden, stifling innovative gains and corporate innovation investments [17, 22, 23]. Consequently, they act as an impediment to the further growth of first-tier cities. In summary, deviations in housing prices from their fundamental values influence cities differently across tiers. Thus, governmental regulatory policies for real estate should be tailored to local conditions.

### 5.4 Endogeneity test and robustness tests

#### 5.4.1 Endogeneity test

This paper addresses potential endogenous issues concerning macroeconomy and housing prices’ deviation from the basic price. Endogeneity mainly comes from three aspects: measurement error, omitted variables, and the reverse causality between independent variables and dependant variables. In t the benchmark model, we add some controlled variables, which including temporal virtual variable and time trend item, to control the impacts of the economic cycle. Urban-level virtual variables and feature variables are added to the controlled variables to control the impacts of certain unobservable individual features. Individual fixed effects, time trend item and time fixed effects have solved the issues of omitted variables and measurement error to a certain extent.

However, the degree of house price’s deviation from the fundamental value may be of reciprocal causation with the dependant variable. To tackle potential endogeneity issues more appropriately, we consulted the research of James [57] and adopted his method of using the Bartik instrumental variable (IV) for endogeneity correction. We considered two main types of real estate: residential and commercial, and employed the interaction of their segmented market characteristics with external price growth rates to construct the Bartik IV. The specific steps are: (1) Firstly, we computed the share of residential and commercial real estate in the total real estate for each city during the baseline year, providing us a benchmark reflecting the local real estate market structure. (2) Subsequently, we calculated the average price growth rate of other cities separately for residential and commercial real estate. This "external" growth rate offers a predictive tool, estimating how real estate prices in a given city would change with external market variations, ensuring that this estimation is not influenced by potential endogenous factors in our study target. (3) Lastly, combining the real estate structure from the baseline year and external growth rates derived from other cities, we projected the change in real estate values for each city. This expected change serves as our constructed Bartik IV, playing a pivotal role in subsequent causal relationship analyses.

The data for commercial and residential sales areas, as well as the average selling prices, are sourced from the WIND database and the National Bureau of Statistics website. Table 15 presents the regression results using the Bartik instrumental variable based on the baseline model. In the first-stage estimation, the results highlight a significant positive correlation between the instrumental variable and the housing bubble index. With an F-statistic exceeding 20, there’s a strong indication that the weak instrument problem is not present. In the second-stage estimation, the findings reveal a significant negative impact of the housing bubble on consumption, GDP, real economic output, and investment. These results are consistent with those from the baseline regression, underscoring the robustness of our baseline estimates.

**Table 15:** Endogeneity test.

Variables	Consumption	Real Economy Investment	Real Economy Output	GDP
Panel A:2SLS regressions				
Housing Bubble Index	-0.081***	-0.409***	-0.266***	-0.139***
	(0.011)	(0.147)	(0.093)	(0.049)
Intercept	18.416***	3.155***	10.482***	15.322***
	(0.060)	(0.986)	(0.486)	(0.872)
Control Variables	YES	YES	YES	YES
Time Trend	YES	YES	YES	YES
Time Effect	YES	YES	YES	YES
Individual Effect	YES	YES	YES	YES
Observations	466	415	229	226
R-squared	0.955	0.952	0.979	0.989
Number of Clusters	35	35	35	35
Panel B:2SLS first-stage regressions (Bartik instrument)				
Bartik index	0.579***	0.668***	0.174***	0.385***
	(0.065)	(0.101)	(0.075)	(0.089)
F-value	26.225	16.866	21.358	14.733

#### 5.4.2 Robustness test

Since there might be a auto-correlation for the housing price bubble index in the temporal dimension, we adopt the lag item as the instrumental variable and run GMM regression. The regression results are listed in Table 16. Since the first-tier cities’ housing price bubble index is much larger than that of other cities, we drop the first-tier cities from the sample. The empirical results are showed in Table 17. Meanwhile, we have adopted the random effect model for empirical examination, and the results are shown in Table 18. Comparing Table 16 with the benchmark regression results (Tables 3 and 6) and heterogeneous regression results (Tables 7 and 12), we discover that key variables’ coefficients show the same trend as that of the benchmark regression results, thus implied that the regression results are robust.

**Table 16:** Robustness test (GMM estimation).

Variables	Consumption	Substantial economic investment	Substantial economic output	GDP
Housing price bubble index	-0.0723***	-0.193***	-0.620***	-0.130***
	(0.0154)	(0.0576)	(0.103)	(0.0305)
Intercept term	17.80***	3.209	10.63***	16.09***
	(0.0303)	(2.919)	(0.491)	(0.565)
Controlled Variables	YES	YES	YES	YES
Time trend	YES	YES	YES	YES
Temporal effects	YES	YES	YES	YES
Individual effects	YES	YES	YES	YES
Observed Value	465	428	234	235
R-squared	0.995	0.938	0.982	0.996
Clustering number	35	35	35	35

**Table 17:** Robustness test (First-tier city omitted).

Variables	Consumption	Substantial economic investment	Substantial economic output	GDP
Housing price bubble index	-0.0486**	-0.185**	-0.385***	-0.0637**
	(0.0184)	(0.0670)	(0.0737)	(0.0292)
Intercept term	15.08***	-230.6***	-386.9***	-249.6***
	(0.0388)	(9.321)	(22.50)	(7.069)
Controlled Variables	YES	YES	YES	YES
Time trend	YES	YES	YES	YES
Temporal effects	YES	YES	YES	YES
Individual effects	YES	YES	YES	YES
Observed Value	441	462	221	491
R-squared	0.986	0.913	0.930	0.976
Clustering number	29	29	29	29

**Table 18:** Robustness test (Random effect).

Variables	Consumption	Substantial economic investment	Substantial economic output	GDP
Housing price bubble index	-0.0446**	-0.243***	-0.289***	-0.0441*
	(0.0196)	(0.0418)	(0.0645)	(0.0242)
Intercept term	15.11***	-139.9***	-381.7***	-249.1***
	(0.167)	(45.03)	(22.14)	(6.855)
Controlled Variables	YES	YES	YES	YES
Time trend	YES	YES	YES	YES
Temporal effects	YES	YES	YES	YES
Individual effects	YES	YES	YES	YES
Observed Value	475	431	236	525
Clustering number	35	35	35	35

## 6. Conclusions

In this paper, using the stochastic frontier model, we accurately measured the fundamental prices in real estate and subsequently computed the housing bubble index for the primary cities nationwide. Additionally, this paper empirically tested the impact of the housing price bubble on residents’ consumption, real economic output, real economic investment, and total output. The primary findings are as follows: Firstly, the phenomenon of housing price bubbles is prevalent. Research indicates that most cities in China exhibit signs of a housing price bubble, with first-tier cities showing a more pronounced level. This discovery aligns with the research results of Wang & Liu [3] and Meng et al. [47], further confirming the bubble phenomenon in China’s real estate market. Secondly, housing price bubbles have a detrimental impact on the macroeconomy. Research suggests that housing price bubbles lead to decreased consumption by residents, crowding out investment and production levels in the real economy. This observation is consistent with the studies by Yan & Zhu [29], Luo & Zhang [21], and Chen [39], revealing the potential threats of housing price bubbles to the macroeconomy. Lastly, the effects of housing price bubbles evolve with stages of economic development. As shown, with China’s economic progression, the stimulatory and crowding-out effects of housing bubbles wax and wane, respectively. In the early stages of China’s economic development, housing bubbles primarily served to stimulate economic growth. However, with the gradual elevation of urbanization and industrialization, the crowding-out effects of housing bubbles become more evident. This insight offers empirical support for the studies by Wang & Lu [58], emphasizing the importance of government adjustments to macro-control policies during different economic development stages.

Based on the findings of this study, we propose the following policy recommendations:

1. Tailored Policies for Different Cities to Enhance Real Estate Regulation Efficiency. Given the significant disparities in housing bubble levels across Chinese cities, local governments should formulate regulatory policies by considering the local economic development level and housing bubble index. They should flexibly employ policies like purchase restrictions, interest rates, land supply, and more, tailoring solutions to specific cities and avoiding a one-size-fits-all approach. This will more effectively alleviate the urban housing bubble issue, thereby enhancing the efficiency and effectiveness of real estate regulation.
2. Beware of the Negative Impacts of Housing Bubbles and Avoid Relying on Real Estate for Short-Term Economic Growth Stimulation. Housing bubbles not only lead to decreased consumer spending but also crowd out investment and output in the real economy. Hence, governments shouldn’t blindly pursue short-term growth from a booming real estate market while neglecting the negative effects of housing price bubbles. Especially during the post-pandemic economic recovery phase, the Chinese government should cautiously adjust macro-control measures for real estate, preventing excessive speculative behavior in the housing market and the resurgence of housing bubbles.
3. Given the Evolving Stimulatory and Crowding-out Effects of Housing Bubbles with Economic Structure Shifts, the Government Should Promote Economic Growth Transition and Optimize Industrial Structure. This entails reducing excessive dependence on real estate, enhancing the economy’s intrinsic momentum, and innovative capabilities. Specifically, the government should support technological innovation and the growth of high-end manufacturing to elevate the economy’s added value and competitiveness, develop the service and consumer goods sectors to expand the domestic market’s size and potential, and improve human capital and educational investment to increase the labor market’s supply and quality".

In recent years, the Chinese government has implemented the "housing for living, not for speculation" policy in the real estate market. The findings of our study provide significant empirical support for this policy. Firstly, the policy aims to stabilize and curb speculative demand and irrational behaviours in the housing market, which in turn helps steady market expectations and suppress overinflated housing bubbles, thereby ensuring the healthy and steady development of the real estate market. Secondly, by stabilizing house prices, the "housing for living, not for speculation" policy is expected to reduce the housing burden on residents, increase their disposable income and consumption capacity, thus helping alleviate the negative impacts of housing price bubbles on the macro economy. Lastly, under the new circumstances of real estate market development, this policy diminishes the speculative nature of housing and is conducive to balancing the development of the real estate and the real economy, thereby promoting economic structural transformation, growth momentum shift, and industrial structure optimization.

The principal contributions of this paper are the following: Firstly, we adopted the Stochastic Frontier Model to decompose real estate prices in large and medium-sized cities in China, resulting in a housing price bubble index. This approach is more comprehensive and scientifically rigorous than previous methods that relied on simple indicators like the house-price-to-income ratio or the rent-to-price ratio to gauge housing bubbles. It can more accurately reflect the level of housing bubbles in China. Secondly, based on the calculated housing bubble index, we constructed a panel fixed-effects model using city-level data, empirically testing the impact of housing bubbles on macroeconomic variables. This contributes to unveiling the mechanism through which housing bubbles affect the macroeconomy. Moreover, we also examined the effect of housing bubbles on the macroeconomy at different stages of economic development. The study found that the positive and negative effects of housing bubbles on the macroeconomy might change as the economic structure evolves. This insight helps us understand the shift in China’s economic growth drivers and the necessity and urgency of structural adjustments.

However, our research also has room for improvement. Future studies could refine the methods used to measure housing bubbles, such as employing multiple decomposition techniques for comparative research, to bolster the robustness of the decomposition results of real estate prices. In terms of data selection, a more comprehensive city sample could be used, providing a more holistic view of China’s housing bubble landscape. Methodologically, future research could establish a theoretical model to delve deeper into how housing bubbles impact the macroeconomy, clarifying the underlying mechanisms.

## Supporting information

S1 Data (XLSX)
