# Expression of Concern: Do high house prices promote the development of China’s real economy? Empirical evidence based on the decomposition of real estate price

**DOI:** 10.1371/journal.pone.0334267

**Published:** 2025-10-13

**Authors:** The PLOS One Editors

The *PLOS One* Editors issue this Expression of Concern due to concerns about the article’s [1] peer review and compliance with the PLOS Authorship policy.

In addition, the following issues were identified:

- The undeclared use of a generative AI tool during the manuscript revisions process.
- A lack of supporting citations for statements in the Introduction section.

The authors declared that ChatGPT-4.0 was used for language polishing, and they stated that they verified for the scientific accuracy of AI-revised content. The *PLOS One* Editors consider this issue resolved.

The authors acknowledged the lack of citations in the Introduction section and explained that a ‘background-style’ Introduction without literature citations followed by a cited Literature Review is a common manuscript format in Chinese-language journals.
